# Editorial: Integrative Approaches to Analyze Cancer Based on Multi‐Omics

**DOI:** 10.3389/fgene.2022.1057408

**Published:** 2022-10-17

**Authors:** Sipeng Shen

**Affiliations:** ^1^ Department of Biostatistics, Center for Global Health, School of Public Health, Nanjing Medical University, Nanjing, China; ^2^ Jiangsu Key Lab of Cancer Biomarkers, Prevention and Treatment, Jiangsu Collaborative Innovation Center for Cancer Personalized Medicine, Nanjing Medical University, Nanjing, China; ^3^ Key Laboratory of Biomedical Big Data of Nanjing Medical University, Nanjing, China

**Keywords:** cancer, trans-omics, GWAS—genome-wide association study, bioinformatics and computational biology, next-generation sequencing

Cancer is a multifactorial malignant disease driven by environmental exposure, genetic polymorphism, somatic mutation events, and other downstream omics (Shen et al., 2021a; Sung et al., 2021). In the era of big data, leveraging high dimensional omics data and conducting computational studies can advance oncogenomics research. Integration of multi-omics tumor profiling data, supported by compatible algorithms, enables the establishment of novel cancer biomarkers and personalized treatment strategies aimed at reducing cancer-specific death and improving patient prognosis (Akhoundova and Rubin, 2022). Moreover, with the development of multi-omics designed studies, large-scale and high-quality omics databases are gradually established and open to the public (Table 1). While the omics data cost huge, most of the research articles on our topic leveraged publicly available data (e.g., The Cancer Genome Atlas) and made certain discoveries.

**TABLE 1:** Introduction of public databases with available pan-cancer omics data.

Database	Omics data	Sample size	Feature	URL
UK Biobank	Genomics, metabolomics, proteomics	≈500,000	Natural population cohort	https://www.ukbiobank.ac.uk/
The Cancer Genome Atlas (TCGA)	Genomics, transcriptomics, epigenomics, proteomics	≈10,000	Pan-cancer cohort with large sample size	https://portal.gdc.cancer.gov/
Clinical Proteomic Tumor Analysis Consortium (CPTAC)	Proteomics, genomics, transcriptomics, epigenomics	≈1,500	Pan-cancer cohort with high quality proteomics data	https://proteomics.cancer.gov/programs/cptac
The Pan-Cancer Analysis of Whole Genomes (PCAWG)	Genomics, transcriptomics	≈2,700	Pan-cancer cohort with whole genome sequencing data	https://dcc.icgc.org/pcawg/
FinnGen	Genomics	≈300,000	Natural population cohort	https://www.finngen.fi/en
Gene Expression Omnibus	Transcriptomics, epigenomics	—	Data uploaded when the article is published	https://www.ncbi.nlm.nih.gov/geo/
dbGAP	Genomics	—	Data uploaded when the article is published	https://dbgap.ncbi.nlm.nih.gov/
TARGET	Genomics, transcriptomics, epigenomics	≈6,000	Focus on childhood cancers	https://portal.gdc.cancer.gov/
Research Program on Genes, Environment and Health (RPGEH)	Genomics	≈78,000	Natural population cohort	https://divisionofresearch.kaiserpermanente.org/genetics/rpgeh
MSK (MSK-IMPACT, MSK-CH, MSK-MET)	Genomics	≈25,000	Target sequencing data of somatic events, clonal hematopoiesis, and metastatic events and tropisms	http://www.cbioportal.org/

The large-scale cancer omics studies greatly promote the research of tumor etiology, progression, outcome, and treatment. The first glorious achievement is the identification of numerous cancer-related loci through genome-wide association studies (GWAS) (Tam et al., 2019). As the sample size increases with sufficient statistical power, causal single nucleotide polymorphisms (SNPs) have been reported for major cancers. However, the mechanistic gap between variants and traits is still hard to bridge, while the majority of the identified variants are located in non-coding regions and have been shown to have limited functions (Wu et al., 2018). Thus, it is essential to link the genetic variants to downstream omics to explain the biological functions. The first approach is leveraging the current *in-silico* databases to perform functional annotation analyses, such as expression, splice, methylation, metabolite, protein quantitative trait locus (QTL), histone modification, and protein-bound. The second approach is to predict trans-omics biomarkers based on QTL information and then evaluate the association of predicted biomarkers and cancer outcomes, such as transcriptome-wide association (TWAS) (Gusev et al., 2016) and Mendelian randomization (MR) (Zheng et al., 2020). These post-GWAS studies support the findings of GWAS and provide favorable evidence for exploring the relationship between multi-omics markers and cancers.

The second glorious achievement is the development of biotechnology and bioinformatics approaches to understand multi-omics data, including genomics, transcriptomics, epigenomics, metabolomics, and proteomics. They have updated our understanding of oncology and improved the accuracy of outcome prediction.

In genomics, somatic mutation events (e.g., point mutation, tumor mutation burden, rearrangements) derived from tumor tissues and matched normal tissues in next-generation sequencing (NGS) give us novel insights into tumor driver factors and are practical to guide clinical therapy, such as targeted therapy and immunotherapy. In transcriptomics, RNA sequencing of bulk and single-cell technology advances us to understand the various RNA biomarkers that play essential roles in tumor regulation, proliferation, differentiation, and metastasis (Zhang et al., 2022). While the protein-coding genes have been deeply investigated, the function of non-coding RNAs remains largely unknown, such as long non-coding RNA (lncRNA), circular RNA (circRNA), and PIWI-Interacting RNA (piRNA) (Shen et al., 2021b). Studies have found that non-coding RNAs had a close relationship with tumor microenvironment, immune checkpoints, and specific mechanisms, such as N6-Methyladenosine, ferroptosis, and autophagy (Sun et al.; Zhao et al., Lan et al., Yang et al.). In epigenomics, epigenetic modifications play important roles in the DNA chromatin structure and accessibility, affecting gene transcription and regulation. Among these, DNA methylation marks at the cytosine-phosphate-guanine (CpG) dinucleotide sites are extensively documented that regulate gene expression, genome stability, and cell fate (Shen et al., 2018). Numerous successful epigenome-wide association studies (EWAS) have discovered important CpG sites across human diseases (Campagna et al., 2021). In addition, mass spectrometry (MS)-based proteomics and metabolomics are downstream biomarkers with remarkable effects on cancer outcome, which could reflect the cancer course more directly and should be paid more attention (Lotta et al., 2021; Satpathy et al., 2021).

For multi-omics data, various types of integration methods and algorithms are proposed, which could be generally classified into two fields: traditional methods and artificial intelligence (AI). The traditional statistical methods and bioinformatic algorithms are widely recognized. For example, Shen et al. (2017). performed variable selection based on DNA methylation using sure independence screening (SIS) and developed a trans-omics prognosis model including CpG sites and their corresponding gene expression based on Cox proportional hazards model to predict the overall survival of oral squamous cell carcinoma. The integrated model of clinical characteristics, methylation, and gene expression outperformed single omics. Moreover, bioinformatic methods are practical, such as gene co-expression network, unsupervised similar omics network fusion, pathway enrichment analysis, gene set variation analysis (Shen et al., 2019). Recently, AI is becoming a hotspot where machine learning and deep learning are widely applied in diagnosis and risk/prognosis prediction using cancer omics data (Arjmand et al.). AI generally has higher accuracy for cancer diagnosis and prediction, while it could consider the complex high-order interaction effects ignored in parametric statistical models. However, an enormous disadvantage of AI is the “black box” problem that it does not consider causal medical relationships and could not explain the potential pathogenesis mechanism.

However, challenges still exist for trans-omics studies. First, large-scale DNA sequencing [e.g., whole exome sequencing (WES), whole genome sequencing (WGS)] is gradually focused on for its high coverage of genetic variants. For example, the UK Biobank 150 k WGS project contains 585 million single nucleotide variants (SNVs). At the same time, most of them are rare variants (minor allele frequency <0.01) and ultra-rare variants (minor allele carrier <10), which should not be ignored and might explain part of “missing heritability” (Halldorsson et al., 2022). However, current QTL databases could not contain all rare variants that need novel methods to explore the trans-omics biomarkers, such as variant set-based design. Second, most prediction models only focus on the performance (e.g., Area Under Curve, C-index) but ignore the causal biological relationship (Shu et al.; Zhou et al.). Nonetheless, the mechanism should be comprehensively understood for adjuvant treatment and drug development to seek valuable and practical target therapy biomarkers. Third, although the definition of omics data is well established, deep data-mining of omics data is still insufficient. In addition, new biotechnological (e.g., single-cell sequencing, radiomics, electronic medical records) and computational methods (e.g., deep learning, natural language processing) have been developed, both of which require further research.

In conclusion, trans-omics tumor investigation approaches have rapidly developed, diving deeply into the molecular landscapes of tumors, and elucidating exciting novel aspects of cancer biology. Clinical application of multi-omics biomarkers will further improve our understanding of tumor biology and significantly shape cancer precision treatment in the future.

## References

[B1] Akhoundova D. Rubin M. A. (2022). Clinical application of advanced multi-omics tumor profiling: Shaping precision oncology of the future. Cancer Cell 40 (9), 920–938. 10.1016/j.ccell.2022.08.011 36055231

[B2] Campagna M. P. Xavier A. Lechner-Scott J. Maltby V. Scott R. J. Butzkueven H. (2021). Epigenome-wide association studies: Current knowledge, strategies and recommendations. Clin. Epigenetics 13 (1), 214. 10.1186/s13148-021-01200-8 34863305 PMC8645110

[B3] Gusev A. Ko A. Shi H. Bhatia G. Chung W. Penninx B. W. (2016). Integrative approaches for large-scale transcriptome-wide association studies. Nat. Genet. 48 (3), 245–252. 10.1038/ng.3506 26854917 PMC4767558

[B4] Halldorsson B. V. Eggertsson H. P. Moore K. H. S. Hauswedell H. Eiriksson O. Ulfarsson M. O. (2022). The sequences of 150, 119 genomes in the UK Biobank. Nature 607 (7920), 732–740. 10.1038/s41586-022-04965-x 35859178 PMC9329122

[B5] Lotta L. A. Pietzner M. Stewart I. D. Wittemans L. B. L. Li C. Bonelli R. (2021). A cross-platform approach identifies genetic regulators of human metabolism and health. Nat. Genet. 53 (1), 54–64. 10.1038/s41588-020-00751-5 33414548 PMC7612925

[B6] Satpathy S. Krug K. Jean Beltran P. M. Savage S. R. Petralia F. Kumar-Sinha C. (2021). A proteogenomic portrait of lung squamous cell carcinoma. Cell 184 (16), 4348–4371.e40. e4340. 10.1016/j.cell.2021.07.016 34358469 PMC8475722

[B7] Shen S. Wang G. Shi Q. Zhang R. Zhao Y. Wei Y. (2017). Seven-CpG-based prognostic signature coupled with gene expression predicts survival of oral squamous cell carcinoma. Clin. Epigenetics 9, 88. 10.1186/s13148-017-0392-9 28852427 PMC5571486

[B8] Shen S. Wang G. Zhang R. Zhao Y. Yu H. Wei Y. (2019). Development and validation of an immune gene-set based Prognostic signature in ovarian cancer. EBioMedicine 40, 318–326. 10.1016/j.ebiom.2018.12.054 30594555 PMC6412087

[B9] Shen S. Wei Y. Li Y. Duan W. Dong X. Lin L. (2021). A multi-omics study links TNS3 and SEPT7 to long-term former smoking NSCLC survival. NPJ Precis. Oncol. 5 (1), 39. 10.1038/s41698-021-00182-3 34002017 PMC8128887

[B10] Shen S. Zhang R. Guo Y. Loehrer E. Wei Y. Zhu Y. (2018). A multi-omic study reveals BTG2 as a reliable prognostic marker for early-stage non-small cell lung cancer. Mol. Oncol. 12 (6), 913–924. 10.1002/1878-0261.12204 29656435 PMC5983115

[B11] Shen S. Zhang R. Jiang Y. Li Y. Lin L. Liu Z. (2021). Comprehensive analyses of m6A regulators and interactive coding and non-coding RNAs across 32 cancer types. Mol. Cancer 20 (1), 67. 10.1186/s12943-021-01362-2 33849552 PMC8045265

[B12] Sung H. Ferlay J. Siegel R. L. Laversanne M. Soerjomataram I. Jemal A. (2021). Global cancer statistics 2020: GLOBOCAN estimates of incidence and mortality worldwide for 36 cancers in 185 countries. Ca. Cancer J. Clin. 71 (3), 209–249. 10.3322/caac.21660 33538338

[B13] Tam V. Patel N. Turcotte M. Bosse Y. Pare G. Meyre D. (2019). Benefits and limitations of genome-wide association studies. Nat. Rev. Genet. 20 (8), 467–484. 10.1038/s41576-019-0127-1 31068683

[B14] Wu L. Shi W. Long J. Guo X. Michailidou K. Beesley J. (2018). A transcriptome-wide association study of 229, 000 women identifies new candidate susceptibility genes for breast cancer. Nat. Genet. 50 (7), 968–978. 10.1038/s41588-018-0132-x 29915430 PMC6314198

[B15] Zhang Z. Wang Z. X. Chen Y. X. Wu H. X. Yin L. Zhao Q. (2022). Integrated analysis of single-cell and bulk RNA sequencing data reveals a pan-cancer stemness signature predicting immunotherapy response. Genome Med. 14 (1), 45. 10.1186/s13073-022-01050-w 35488273 PMC9052621

[B16] Zheng J. Haberland V. Baird D. Walker V. Haycock P. C. Hurle M. R. (2020). Phenome-wide Mendelian randomization mapping the influence of the plasma proteome on complex diseases. Nat. Genet. 52 (10), 1122–1131. 10.1038/s41588-020-0682-6 32895551 PMC7610464

